# Porcine epidemic diarrhea virus nucleoprotein contributes to HMGB1 transcription and release by interacting with C/EBP-β

**DOI:** 10.18632/oncotarget.11991

**Published:** 2016-09-13

**Authors:** Chang-chao Huan, Hua-xia Wang, Xiang-xiang Sheng, Rui Wang, Xin Wang, Ying Liao, Qin-fang Liu, Guang-zhi Tong, Chan Ding, Hong-jie Fan, Jia-qiang Wu, Xiang Mao

**Affiliations:** ^1^ College of Veterinary Medicine, Nanjing Agricultural University, Nanjing, Jiangsu Province, China, 210095; ^2^ Shanghai Veterinary Research Institute, Chinese Academy of Agricultural Sciences, Shanghai, China, 200241; ^3^ Institute of Animal Science and Veterinary Medicine, Shandong Academy of Agricultural Sciences, Shandong Province, China, 250100

**Keywords:** porcine epidemic diarrhea virus, HMGB1, nucleoprotein, C/EBP-β

## Abstract

Porcine epidemic diarrhea is a devastating swine enteric disease, which is caused by porcine epidemic diarrhea virus (PEDV) infection. Our studies demonstrated that PEDV infection resulted in the up-regulation of proinflammatory cytokines. Meanwhile, PEDV infection and overexpression of viral nucleoprotein resulted in the acetylation and release of high mobility group box 1 proteins *in vitro*, an important proinflammatory response mediator, which contributes to the pathogenesis of various inflammatory diseases. Our studies also showed that SIRT1, histone acetyltransferase, and NF-κB regulated the acetylation and release of HMGB1. Chromatin immunoprecipitation, dual-luciferase reporter gene assay, and co-immunoprecipitation experiments illustrated that PEDV-N could induce HMGB1 transcription by interacting with C/EBP-β, which could bind to C/EBP motif in HMGB1 promotor region. Collectively, our data indicate PEDV-N contributes to HMGB1 transcription and the subsequent release/acetylation of HMGB1 during PEDV infection.

## INTRODUCTION

Porcine epidemic diarrhea virus (PEDV), an enveloped, single-stranded and positive-sense RNA virus, is the causative agent of porcine epidemic diarrhea (PED) [1]. PEDV belongs to the genus of *Alphacoronavirus*, the family of *Coronaviridae* and the subfamily of *Coronavirinae* [2]. It was originally reported in Belgium and the United Kingdom [3]. PEDV infection causes 80 to 100% fatality rate in suckling piglets [4]. The outbreak of PED in the United States in 2013 caused significant economic loss [5]. In 2014, PED swept across two sow farms and one fattening farm in South-Western Germany [6]. In January 2015, an outbreak of porcine epidemic diarrhea (PED) emerged in Portugal [7]. Currently, several PEDV vaccines are available, further studies on PEDV pathogenesis will be beneficial to the development of new vaccines.

PEDV encodes several structural proteins, including the spike (S), envelope (E), membrane (M), and nucleoprotein (N) [8]. The nucleoprotein of coronaviruses assists the correct folding, packaging and encapsidation of viral RNA. Meanwhile, it can also regulate the host cell cycle to benefit virus infection [9–11]. During the infection, PEDV nucleoprotein protein (PEDV-N) is mainly localized in the cytoplasm, but a lesser amount of it also exists in the nucleus [12]. PEDV-N antagonizes interferon-β production by sequestering the interaction between IRF3 and TBK1 [13].

Inflammation initiates host defense against infection or injury by activating innate and adaptive responses. Pathogens have evolved different strategies to support their survival by manipulating the host immune responses. Some pathogens evade the immune system and consequently lessen the protective immune response. Meanwhile, other pathogens hyper-stimulate the immune system (cytokine storm) to avoid the clearance of infection and induce tissue damage. Cytokine storm triggered by virus infection is characterized by a significant increase of proinflammatory cytokines (TNF-α, IL-1β, IL-8, and IL-6) that subsequently results in fever, edema, organ dysfunction and even death [14].

Pattern recognition receptors (PRRs) sense microorganisms and induce proinflammatory responses. Except for the recognition of pathogen–associated molecular patterns (PAMPs), PRRs also recognize endogenous molecules that are released during cellular injury or tissue damage, including heat shock and high mobility group box protein 1 (HMGB1) proteins, which are termed as damage–associated molecular patterns (DAMPs) [15]. HMGB1, previously known as HMG-1 or amphoterin, was discovered over 30 years ago [16]. HMGB1 is a highly conserved and ubiquitous DNA-binding protein which functions as a structural protein of chromatin. It localizes in the nuclei of almost all cell types and participates in the maintenance of nucleosome structure and DNA replication [17]. HMGB1 has a tripartite structure: two homologous DNA-binding domains, HMG boxes, and a C-terminal domain rich with aspartic and glutamic acid residues [18, 19]. The biological activities of HMGB1 depend on its location and post-translational modification status. In the nuclei, HMGB1 regulates transcription and recombination through affecting chromosomal architecture [20]. Although HMGB1 does not possess a signal sequence to assist its release through the endoplasmic reticulum/Golgi secretory system, it can be secreted into the extracellular space actively or passively by various cells [21–23]. Extracellular HMGB1 functions as a damage-associated molecular pattern (DAMP) molecule and activates proinflammatory signaling pathways through TLR4 [24, 25] and the receptor for advanced glycation end products (RAGE) [26, 27]. Therefore, HMGB1 is considered as a unique mediator in innate immune responses and inflammation-associated events [28].

In our studies, we first confirmed that PEDV infection increased the expression of proinflammatory cytokines. Virus infection also led to HMGB1 acetylation and release. HMGB1 acetylation and release were regulated by SIRT1, histone acetyltransferase and NF-κB. Further studies showed that the nucleoprotein of PEDV promoted acetylation and release of HMGB1. Chromatin immunoprecipitation and luciferase reporter gene experiments confirmed that C/EBP motif in HMGB1 promotor was critical for HMGB1 transcription. It was further confirmed that PEDV-N protein could initiate HMGB1 transcription via interaction with C/EBP-β.

## RESULTS

### PEDV infection induces the elevation of mRNA levels of proinflammatory cytokines

**Figure 1:**
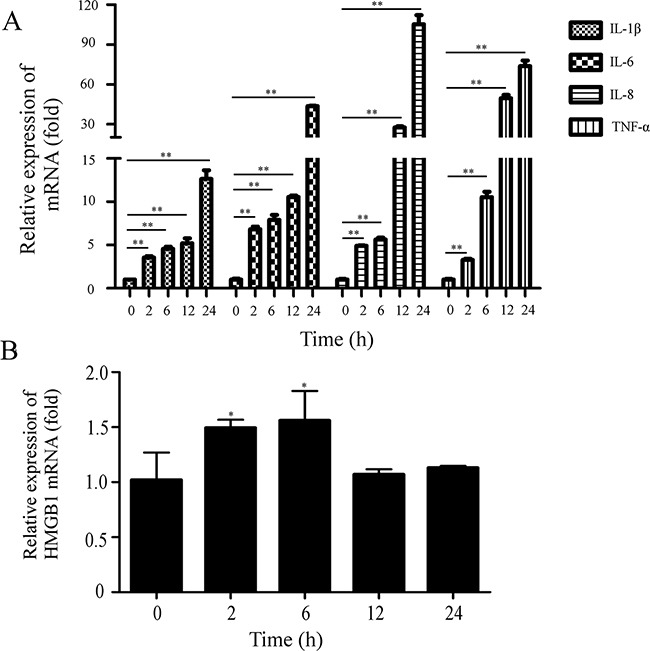
PEDV infection induces the increase of proinflammatory cytokines Vero cells were infected with PEDV (MOI=0.1). The cells were collected at 0, 2, 6, 12, 24hpi. **A.** The mRNA levels of proinflammatory cytokines were analyzed by qRT-PCR. **B.** The mRNA levels of HMGB1 were evaluated by qRT-PCR. The results are the representative of at least two different experiments. The results represent the means ±SD of triplicate determinations. One-way ANOVA; *, P < 0.05; **, P < 0.01.

Although the low amounts proinflammatory cytokines may be protective against viral invasion, they are harmful to the host when overproduced [29]. To explore whether PEDV caused the increase of proinflammatory cytokines, we collected Vero cells infected with PEDV at 2h, 6h, 12h and 24h and measured the mRNA levels of several proinflammatory cytokines and HMGB1. The qRT-PCR result showed that the infection significantly increased the mRNA levels of proinflammatory cytokines TNF-α, IL-1β, IL-6, and IL-8 (Figure 1A); the mRNA levels of cytokine IL-1β, IL-6 and IL-8 were elevated remarkably at 24hpi: IL-1β (~14 folds), IL-6 (~45 folds) and IL-8 (~105 folds), TNF-α (~83 folds). The result implied that PEDV infection might induce aberrant and excessive cytokine production in the cells. Meanwhile, the mRNA level of HMGB1 was weakly increased about 1.5 folds at 2-6 hpi (Figure 1B).

### PEDV infection triggers release of acetylated HMGB1

HMGB1 normally resides in the nucleus [30], but it can migrate from the nucleus to the cytoplasm, as well as to the extracellular space in the activated macrophages/monocytes [31, 32] or in virus-infected cells [33–35].

**Figure 2:**
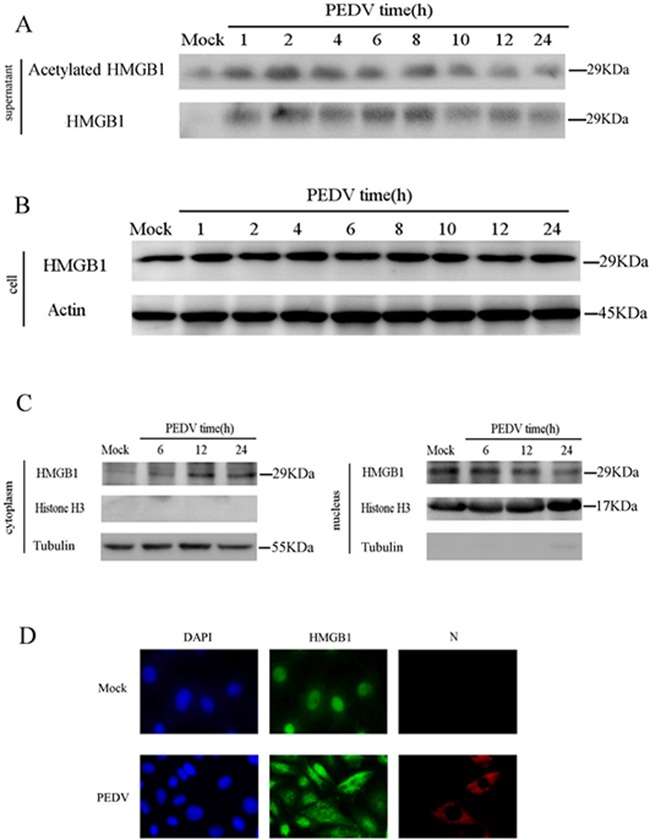
PEDV infection triggers the release of acetylated HMGB1 The protein concentration in the supernatant was determined using the Bradford assay. An equal amount of protein was subject to western blot analysis. **A.** Vero cells were infected with PEDV (MOI=0.1). The supernatant was collected at different time points. Western blot was performed to analyze total and acetylated HMGB1. **B.** HMGB1 in cells was analyzed by western blot at different time points of PEDV infection. **C.** The infected cells were collected at 6h, 12h and 24hpi to analyze HMGB1 protein levels in the cytoplasm and nucleus extracts by western blot. **D.** The translocation of HMGB1 in infected cells at 6 hpi was studied by IFA..

We infected the cells and determined the protein level of HMGB1 in the supernatant at different time points. The data revealed that acetylated and total HMGB1 levels were increased from 1hpi to 8hpi, but decreased later in the supernatant (Figure 2A). PEDV infection barely affected the total HMGB1 level in the cells (Figure 2B), which could be explained by the slight change of mRNA level of HMGB1in the infected cells (Figure 1B). The remarkable stability of HMGB1 has been revealed in previous study [33]. Therefore, we suspected that the posttranslational modification of HMGB1, such as acetylation, might play much more critical roles in the pathogenesis of PEDV infection. We next confirmed the translocation of HMGB1 in infected cells in two experiments. First, the infected cells were collected at 6h, 12h and 24hpi to analyze the protein levels of HMGB1 in the cytoplasm and nucleus extracts separately after nuclear and cytoplasmic fractionation according to instruction of NE-PER™ Nuclear and Cytoplasmic Extraction Kit (Thermo Scientific). The result of western blot analysis suggested that HMGB1 protein expression level was elevated in the cytoplasm, but decreased in the nucleus (Figure 2C). The purity of the fractions obtained was determined by immunoblotting with specific protein markers: histone H3 (nucleus) and tubulin (cytoplasm). Next, the infected cells were stained with a HMGB1 antibody at 6hpi to investigate the localization change of HMGB1. The results demonstrated that HMGB1 was translocated from the nucleus to cytoplasm in most of the infected cells. Additionally, the cells were also immunostained by PEDV-N antibody to confirm the infection (Figure 2D). Overall, our data verified that PEDV infection caused the translocation of HMGB1 from the nucleus to the cytoplasm and release into extracellular space.

### SIRT1, histone acetyltransferase and NF-κB are critical for the acetylation of HMGB1

The acetylation of key lysine residues in HMGB1 is important for its migration and secretion [38, 39]. The proteins in sirtuin family (SIRT1-SIRT7) are class III histone deacetylases (HDACs) that utilize NAD+ as the cofactor [36]. SIRT1 is expressed in most cells, which specifically deacetylates the histone or non-histone proteins. HMGB1 has been reported as one of the deacetylation targets of SIRT1 [37].

**Figure 3:**
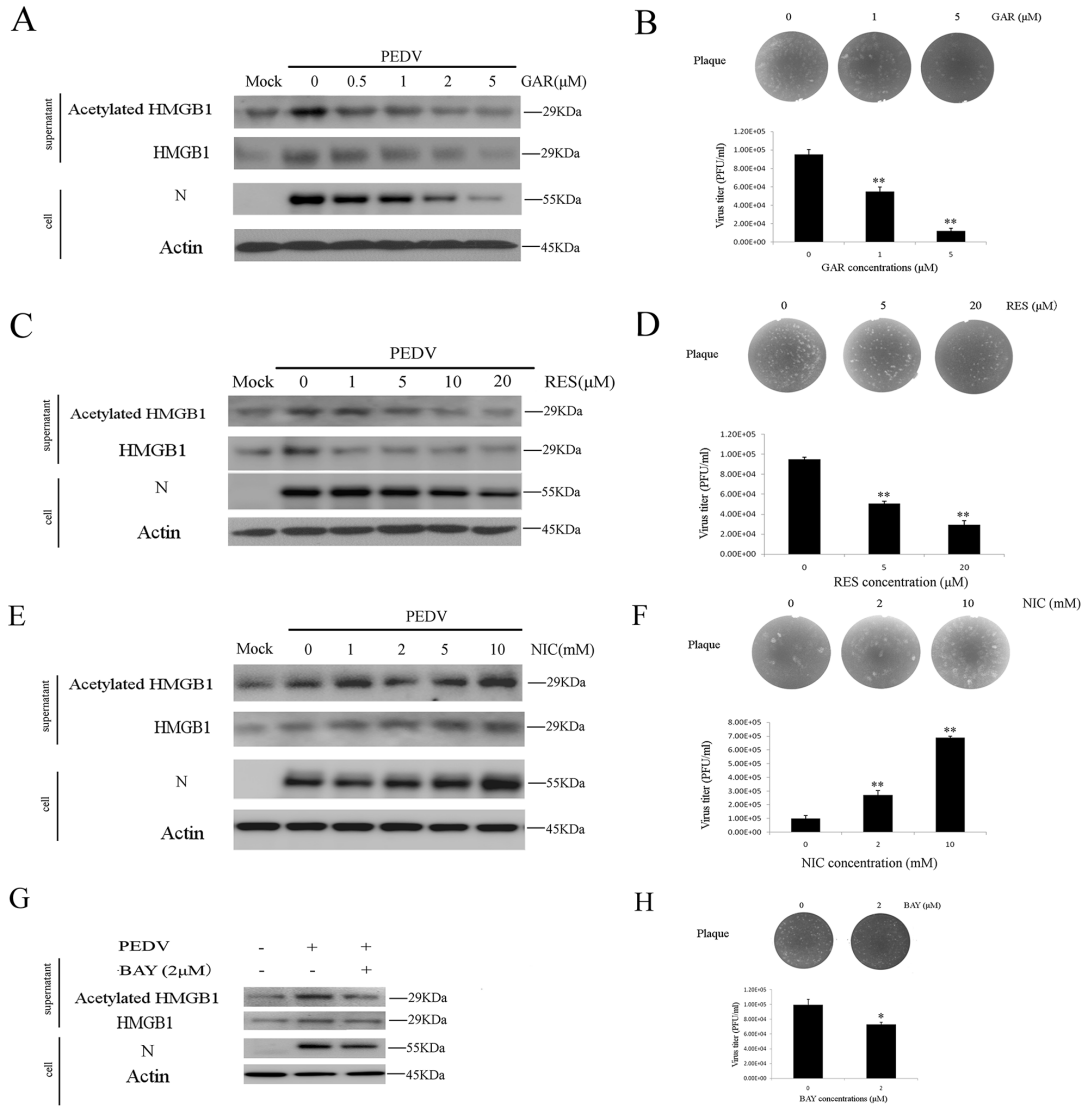
Histone acetyltransferase, SIRT1, and NF-κB are involved in HMGB1 acetylation The protein concentration in the supernatant was determined using the Bradford assay. An equal amount of proteins were used for western blot. **A.** Vero cells were treated with different concentrations GAR (HAT inhibitor) for 1h before infected with PEDV (MOI=0.1) in the presence of different concentrations of GAR for 24h. The acetylated HMGB1, total HMGB1 in the supernatant and PEDV-N in the cells were analyzed by western blot. **B.** The virus titer in the supernatant after GAR treatment was measured by the plaque formation assay. **C.** Vero cells were treated with different concentrations RES (SIRT1 activator) for 1h before infected with PEDV (MOI=0.1) in the presence of different concentrations of RES for 24h. The acetylated HMGB1 or total HMGB1 in the supernatant and PEDV-N in cells were analyzed by western blot. **D.** The virus titer in the supernatant after RES treatment was measured by the plaque formation assay. **E.** Vero cells were treated with different concentrations NIC (SIRT1 inhibitor) for 1h before infected with PEDV (MOI=0.1) in the presence of different concentrations of NIC for 24h. The acetylated HMGB1 or total HMGB1 in the supernatant and PEDV-N in cells were analyzed by western blot. **F.** The virus titer in the supernatant after NIC treatment was measured by plaque formation assay. **G.** Vero cells infected with PEDV (MOI=0.1) were treated with BAY (NF-κB inhibitor) for 24h. The acetylation/release of HMGB1 in the supernatant and PEDV-N in the cells were evaluated by western blot. **H.** The virus titer in the supernatant after BAY treatment was measured by the plaque formation assay. The results are the representative of at least two different experiments. The results represent the means ±SD of triplicate determinations. One-way ANOVA; *, P < 0.05; **, P < 0.01.

Acetylation of HMGB1 is regulated by several histone acetyltransferase (HATs), such as PCAF and P300 [38]. Garcinol (GAR) is a potent HAT inhibitor. We found that GAR treatment reduced the acetylation/release of HMGB1 and PEDV infection in a dose-dependent manner, as suggested by western blotting analysis (Figure 3A). A similar inhibitory trend was observed in the plaque formation assay with the maximum reduction of 85.4% at the concentration of 5 μM (Figure 3B).

We examined the impacts of SIRT1 activator resveratrol (RES) and its inhibitor nicotinamide (NIC) on HMGB1 release and PEDV infection. RES treatment decreased the acetylation/release of HMGB1 significantly, but its effect on the expression level of PEDV-N was only shown at higher concentrations (10 and 20mM) (Figure 3C). On the contrary, NIC treatment promoted HMGB1 acetylation/release and virus infection (Figure 3E). The virus titer in the supernatant was measured by the plaque formation assay respectively. RES treatment decreased the virus titer about 62.3% at the concentration of 20μM (Figure 3D), whereas NIC treatment increased the virus titer about 6.6 folds at the concentration of 10 mM (Figure 3F).

The activation of NF-κB is associated with the acetylation of HMGB1. NF-κB could activate histone acetyltransferases [40]. We, therefore, tested whether NF-κB inhibitor (BAY 11-7082) treatment decreased the acetylation of HMGB1 during PEDV infection. As expected, the inhibitor indeed reduced the acetylation/release of HMGB1, PEDV-N expression level as well as the virus titer (Figure 3G, 3H). All the results confirmed that histone acetyltransferase, SIRT1, and NF-κB were involved in the acetylation of HMGB1.

### PEDV-N promotes the acetylation/release of HMGB1 and expression of proinflammatory cytokines

**Figure 4:**
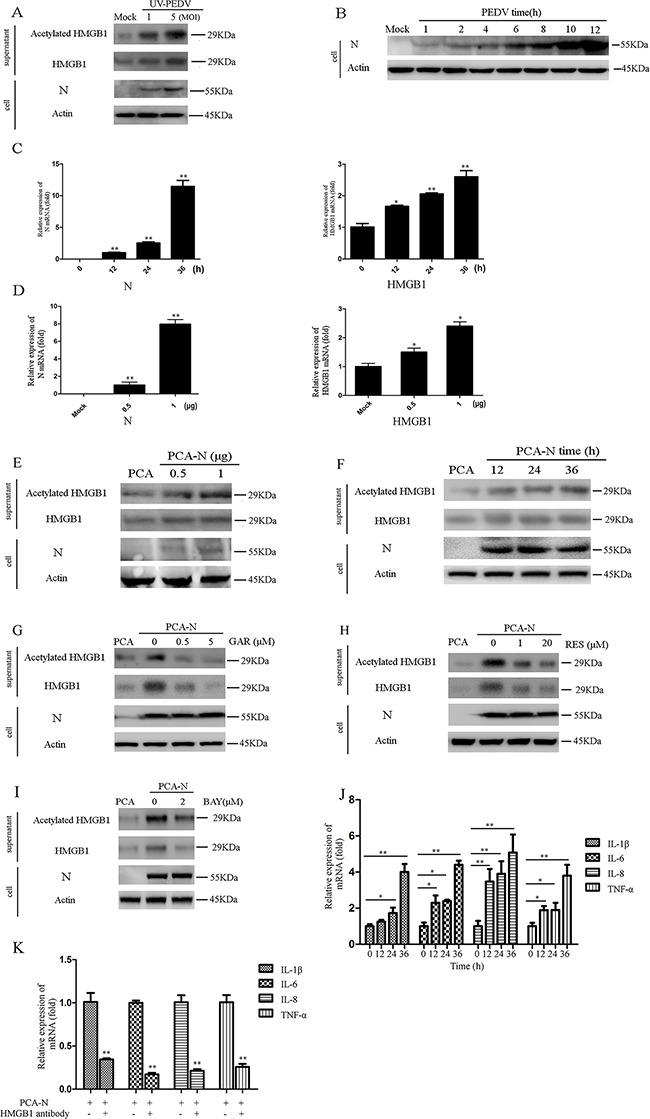
PEDV-N promotes the acetylation/release of HMGB1 and expression of proinflammatory cytokines The protein concentration in the supernatant was determined using the Bradford assay. An equal amount of protein was used for western blot analysis. **A.** Vero cells were infected with UV-inactivated PEDV (UV-PEDV) at different MOI for 1h at 37°C. Total and acetylated HMGB1 in the supernatant were then immediately analyzed by western blot. **B.** Vero cells were infected with PEDV (MOI=0.1). PEDV-N in cells was analyzed by western blot at different time points of the infection. **C.** Vero cells were transfected with PCA-N or PCA (2.5μg) for 12h, 24h, 36h. The mRNA level of HMGB1 or PEDV-N was analyzed by qRT-PCR. **D.** Vero cells were transfected with PCA-N or PCA at different doses (0.5, 1μg) for 36h. The mRNA of HMGB1 or N was analyzed. **E.** and **F.** The acetylated and total HMGB1 in the supernatant were determined by western blot in the cells transfected with PCA-N or PCA at the different amount or time points. The protein concentration in the supernatant was determined using the Bradford assay. An equal amount of protein was used for western blot analysis. **G.** to **I.** Vero cells were first transfected with PCA-N or PCA at the same doses (2.5μg) for 12h and then treated with GAR, RES, or BAY for 24h. The acetylated HMGB1 and total HMGB1 in the supernatant were determined by western blot. **J.** Vero cells were transfected with PCA-N or PCA at the same doses (2.5μg) for 12h, 24h, 36h. The mRNA levels of proinflammatory cytokines were analyzed by qRT-PCR. **K.** Vero cells were transfected with PCA-N or PCA at the same doses (2.5μg) for 12h. The cells were incubated with 1.5μg HMGB1 antibody for 24h. The mRNA levels of proinflammatory cytokines were analyzed by qRT-PCR. The results are the representative of at least two different experiments. The results represent the means ±SD of triplicate determinations. One-way ANOVA; *, P < 0.05; **, P < 0.01.

We next determined whether virus replication was associated with HMGB1 acetylation and release. Vero cells were infected with UV-inactivated PEDV (UV-PEDV) at different MOI (1 and 5) for 1h at 37°C. UV-inactivated virus was able to enter the cells but did not replicate in the cells. The results showed that UV-PEDV infection also induced the release of acetylation HMGB1 in MOI-dependent manner (Figure 4A), suggesting the involvement of virion proteins or genomic RNA in modulating HMGB1 acetylation and release.

The PEDV nucleocapsid (N) protein is an RNA-binding protein, which has vital roles in manipulating host cell processes and viral RNA synthesis. PEDV-N is the most abundant protein in the virion. In PEDV infected cells, N proteins are mainly localized in the cytoplasm, but some portion of it is also localized in the nucleolus [12]. We detected PEDV-N protein in the cells at 1 hpi (MOI=0.1), which could partially come from virion used in the infection (Figure 4B, lane 2). In addition, increase of HMGB1 acetylation and release were observed (Figure 2A). Therefore, we speculated that PEDV-N might be associated with the HMGB1 release.

We next overexpressed the PEDV-N protein in Vero cells to elucidate its effect on HMGB1 expression, acetylation, and release. qRT-PCR result suggested that the increase of mRNA level of PEDV-N was concomitant with an elevated mRNA level of HMGB1 (~2 fold) in a time- and dose-dependent manner (Figure 4C, 4D). To explore whether PEDV-N expression promoted the acetylation and release of HMGB1, the protein level of HMGB1 in the supernatant were also determined by western blotting analysis, which demonstrated that the expression of PEDV-N indeed promoted the acetylation /release of HMGB1 in dose and time-dependent manner (Figure 4E, 4F). Similar to their inhibitory effects on HMGB1 acetylation/release during the infection, GAR, RES, and BAY treatment decreased the acetylation/release of HMGB1 caused by PEDV-N overexpression (Figure 4G, 4H, 4I).

qRT-PCR assay indicated PEDV-N overexpression led to the increase of mRNA levels of proinflammatory cytokines (TNF-α, IL-1β, IL-6, and IL-8), especially at 36h post-transfection (IL-1β: ~4.0 folds, IL-6: ~4.3 folds, IL-8: ~5.1 folds and TNF-α: ~3.9 folds) (Figure 4J). To investigate if released HMGB1 affected the expression of these cytokines, we incubated HMGB1 antibody with PEDV-N overexpressing cells and determined mRNA levels of proinflammatory cytokines. As expected, the upregulation of proinflammatory cytokines induced by PEDV-N overexpression were inhibited (IL-1β: 58%, IL-6: ~82.2%, IL-8: ~78.6%, and TNF-α: ~73%) (Figure 4K). The results strongly suggested that the released HMGB1 indeed induced proinflammatory cytokine production, implying the possible therapeutic function of HMGB1 antibody on aberrant cytokine production.

### PEDV-N is enriched in the HMGB1 promotor and C/EBP binding motif is responsible for HMGB1 transcription

Since PEDV-N promoted HMGB1 release, we next explored whether PEDV-N affected HMGB1 release by interacting with HMGB1. The co-immunoprecipitation experiments were performed using either PEDV-N or HMGB1 antibody. The result indicated that PEDV-N did not interact with HMGB1 (data not shown).

**Figure 5:**
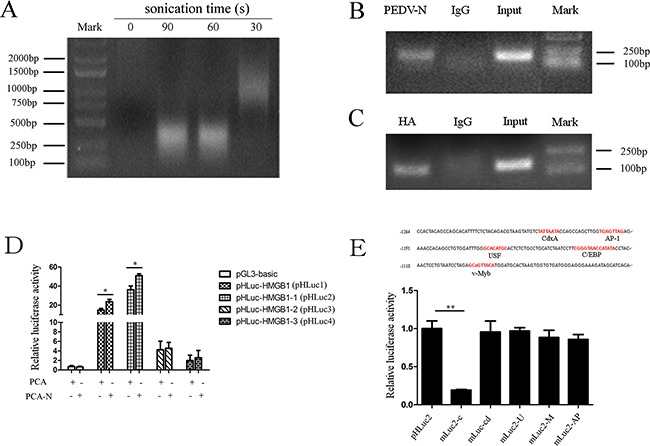
PEDV-N enriches in the HMGB1 promotor and C/EBP binding motif is responsible for HMGB1 transcription **A.** The optimized sonication time in CHIP experiment was explored. **B.** Vero cells were infected with PEDV (MOI=0.1) for 24h. PCR analysis of ChIP samples demonstrated N protein enriched in the HMGB1 gene. **C.** Vero cells were transfected with PCA-N for 24h. PCR analysis of ChIP samples showed HA-tagged PEDV-N protein enriched in the HMGB1 gene. **D.** The reporter gene plasmids containing different HMGB1 promotor region were constructed, including pHLuc-HMGB1 (pHLuc1): −1622 to +83, pHLuc-HMGB1-1 (pHLuc2): −1264 to +83, pHLuc-MGB1-2 (pHLuc3): −822 to +83, and pHLuc-HMGB1-3 (pHLuc4): −382 to +83. Vero cells were cotransfected with each reporter gene plasmid and PCA-N, together with renilla luciferase reporter plasmid pRL-TK for 24h. Empty pGL3-basic and PCA vectors were used as the negative control. Luciferase activity was determined by a dual-luciferase assay system. **E.** Five mutant reporter gene plasmids with transcription factor binding site deletion were constructed, including mLuc2-C for C/EBP, mLuc2-M for v-Myb, mLuc2-AP for AP-1, mLuc2-Cd for CdxA, and mLuc2-U for USF. Vero cells were co-transfected with each mutant reporter gene or WT plasmid (pHLuc2), PCA-N or PCA and renilla luciferase reporter plasmid pRL-TK for 24h. Luciferase activity was determined. Data represented relative firefly luciferase activity normalized to renilla luciferase activity. The results are the representative of at least two different experiments. The results represent the means ±SD of triplicate determinations. One-way ANOVA; *, P < 0.05; **, P < 0.01.

To determine whether HMGB1 genomic DNA was targeted by PEDV-N, we carried out ChIP experiment. Sonication of the chromatin is a critical step in the ChIP experiment, because genomic DNA should be sheared uniformly and randomly. The ideal DNA fragment size after sonication is 200-1000 bp. We found that the most optimized sonication time was 1 min (Figure 5A). ChIP experiment was performed in virus-infected cells or PEDV-N overexpressing cells using anti-N polyclonal antibody or anti-HA monoclonal antibody. The DNA from ChIP experiment was used as the PCR template. A specific PCR product was obtained (Figure 5B and 5C). The PCR products from these two experiments were subsequently ligated into the pUM19-T vector for sequencing. The sequencing results confirmed that the PCR products corresponded to HMGB1 promoter sequence (Figure 5E) [41].

To explore how PEDV-N regulated HMGB1 transcription in Vero cells, HMGB1 promoter region (−1622 to +83) was subcloned into the pGL3-Basic vector (Promega). At the same time, the reporter plasmids containing different promotor regions were also constructed: pHLuc1 (−1622 to +83), pHLuc2 (−1264 to +83), pHLuc3 (−822 to +83), pHLuc4 (−382 to +83). Vero cells were co-transfected together with one of these reporter gene plasmids (pGL3-basic as the control) and pCAGGS-HA-N (PCA-N) or pCAGGS-HA (PCA, empty vector as the control) for 24h before luciferase activities were determined. The result revealed that PEDV-N only increased the transcription of pHLuc2 (−1264 to +83) and pHLuc1 carrying the full-length promoter region (Figure 5D), but not the transcription of pHLuc3 which lacked the promoter region from −1264 to −822. The data strongly suggested that the promoter region from −1264 to −822 was vital for HMGB1 gene transcription (Figure 5D) induced by PEDV-N.

We searched the transcription factor binding sites in the region from −1264 to −822 in TRANSFAC database. It was shown that the putative binding sites for C/EBP, CdxA, AP-1, USF, and v-Myb were located in this promoter region (Figure 5E). We next constructed five mutant reporter gene plasmids by deleting these binding sites one by one (mLuc2-C for C/EBP, mLuc2-M for v-Myb, mLuc2-AP for AP-1, mLuc2-Cd for CdxA, and mLuc2-U for USF). The mutant plasmid was co-transfected with PCA-N. Luciferase assay showed that deletion of C/EBP-binding motif (mLuc2-C) resulted in 75% decrease in luciferase activity, compared with that of wild-type pHLuc2 (Figure 5E). The luciferase activities of the other mutations did not differ significantly from that of pHLuc2. In summary, our data revealed that C/EBP-binding motif in HMGB1 promotor region was vital for its transcription promoted by PEDV-N protein.

### PEDV-N interacts with C/EBP-β

CCAAT/enhancer-binding protein (C/EBP) is an important transcriptional factor that belongs to the leucine zipper family. C/EBP regulates the transcription of a number of genes via protein-protein interaction. C/EBP-β is one of the important members of C/EBP. C/EBP-β can physically interact with other C/EBP proteins, such as Fos and Jun [42]. In addition, C/EBP-β forms complexes with other transcription factors such as NF-κB [43]. C/EBP-β plays important roles during the infection of diverse viruses, including HIV [44], SIV [45], HPV [46], KSHV [47] and HCV [48]. C/EBP-β is also involved in host innate immunity by regulating the expression of TNF-α, IL-1β, IL-10 and IL-8 [49].

**Figure 6:**
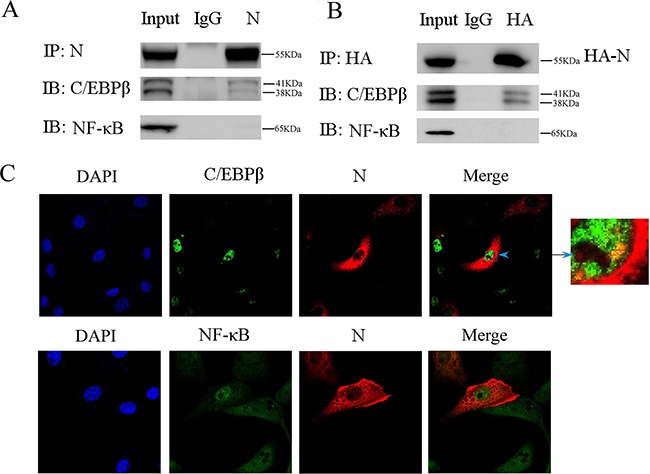
Co-immunoprecipitation experiment reveals that PEDV nucleoprotein interacts with C/EBP-β **A.** Vero cells were infected with PEDV (MOI=0.1) for 24h. The cells were lysed with the lysis buffer containing DNase (100μg/ml) and RNase A(100μg/ml). Immunoprecipitation (IP) experiment was performed with anti-N antibody or control rabbit immunoglobulin G (IgG), followed by immunoblot analysis (IB) with C/EBP-β or NF-κB antibody. **B.** Vero cells were first transfected with PCA-N for 24h. Immunoprecipitation (IP) experiment was performed with anti-HA antibody or control rabbit immunoglobulin G (IgG), followed by immunoblot analysis (IB) with C/EBP-β or NF-κB antibody. **C.** The colocalization between C/EBP-β, NF-κB and PEDV-N was studied under confocal microscopy.

Since our study showed the promotor region containing C/EBP-binding motif was critical for HMGB1 transcription induced by PEDV-N, we next explored the possible interaction between C/EBP-β and PEDV-N with co-immunoprecipitation experiment. Vero cells were infected with PEDV for 24h. The cells were lysed with the lysis buffer containing DNAase and RNase A. The co-immunoprecipitation experiment was performed with PEDV-N antibody, which revealed that PEDV-N interacted with C/EBP-β (Figure 6A). Co-immunoprecipitation experiment was also performed in Vero cells transfected with plasmid PCA-N. The expressed N protein was immunoprecipitated with anti-HA monoclonal antibody and specific interaction with C/EBP-β was also observed (Figure 6B). As a control, PEDV-N did not interact with NF-κB in both experiments (Figure 6A, 6B). In addition, our confocal microscopy experiment confirmed that PEDV-N was colocalized with C/EBP-β in the nucleus, but not with NF-κB (Figure 6C).

### Blockage of HMGB1 inhibited PEDV infection

**Figure 7:**
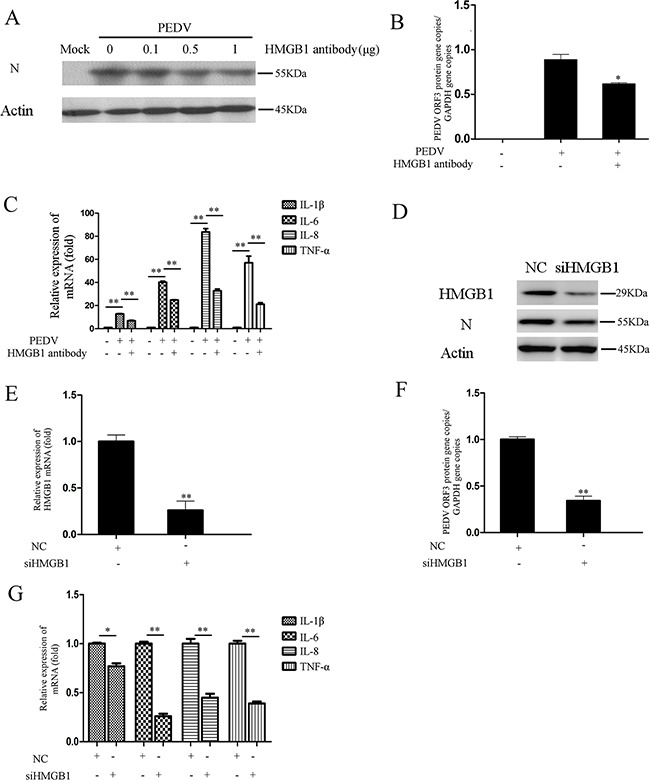
Blockage of HMGB1 inhibits PEDV infection **A.** Vero cells were infected with PEDV (MOI=0.1) and then treated with different concentrations HMGB1 antibody for 24h. The PEDV-N was analyzed by Western blot. **B.** The RNA level of viral ORF3 gene was evaluated in infected cells treated with HMGB1 antibody by qRT-PCR. **C.** The mRNA levels of proinflammatory cytokines (IL-1β, IL-6, IL-8, TNF-α) in infected cells treated with HMGB1 antibody treatment were evaluated by qRT-PCR. **D.** Vero cells were transfected with siHMGB1 to knockdown HMGB1 expression for 24h. siNC was used as the negative control. The cells were infected PEDV for 24h. The protein levels of HMGB1 and PEDV-N were analyzed by western blot. **E.** and **F.** The RNA levels of HMGB1 and PEDV ORF3 genes were measured by qRT-PCR. **G.** The mRNA levels of proinflammatory cytokines (IL-1β, IL-6, IL-8, TNF-α) in infected cells were evaluated by qRT-PCR after HMGB1 expression was knocked down. The results are the representative of at least two different experiments. The results represent the means ±SD of triplicate determinations. One-way ANOVA; *, P < 0.05; **, P < 0.01.

It is well-known that HMGB1 released into the extracellular space can elicit potent inflammatory responses [21]. The above experiments revealed that HMGB1 was actively released into extracellular space during PEDV infection. HMGB1 antibody could decrease the upregulation of proinflammatory cytokine induced by PEDV-N overexpression (Figure 4K). Therefore, we performed antibody neutralization experiment to investigate whether the infection could be inhibited after the HMGB1 protein was neutralized. Vero cells were infected with PEDV and then incubated with HMGB1 antibody for 24h. The western blotting analysis demonstrated that PEDV-N protein expression level was decreased (Figure 7A). Meanwhile, the RNA level of PEDV ORF3 gene was also inhibited by ~27% (Figure 7B) as well as the mRNA levels of the proinflammatory cytokines (IL-1β: ~47.3%, IL-6: 38.1%, IL-8: ~56.9%, TNF-α: 65.6%) after HMGB1 antibody treatment (Figure 7C).

siRNA knockdown experiment was performed to confirm the effect of HMGB1 on PEDV infection. As expected, PEDV-N protein expression was decreased and the RNA level of PEDV ORF3 was decreased 61.5%. The knockdown efficiency of HMGB1 was about 73% (Figure 7D, 7E, 7F). At the same time, the mRNA levels of the proinflammatory cytokines were significantly decreased, especially IL-6 (~74.3%), IL-8 (~53.7%), and TNF-α (~58.8%) (Figure 7G). In summary, our data revealed that blockage of HMGB1 could attenuate PEDV infection and decrease the expression of proinflammatory cytokines.

## DISCUSSION

Host pattern-recognition receptors (PRRs) recognize non-self molecules, such as viral RNA or structural proteins of the virion. After recognition, the infected cell rapidly initiates an innate immune response by inducing antiviral and proinflammatory cytokines expression to restrict viral replication and regulate adaptive immune responses. Although antiviral and proinflammatory cytokines are vital to controlling viral infection, they also cause harmful inflammation and tissue damage which must be tightly controlled. Many viruses, including West Nile virus, SARS-CoV, and hepatitis (A, B, C) viruses induce inflammatory responses by the production of various proinflammatory cytokines. In our studies, we revealed that PEDV infection also resulted in the significant increase of proinflammatory cytokine at mRNA level.

Our study further showed that PEDV infection resulted in the acetylation and release of HMGB1, one of the best known DAMP molecules which can be released by damaged or stressed cells [50]. DAMP molecules lead to the early innate and adaptive immune inflammation after binding to their receptors. HMGB1 contributes to the pathogenesis of influenza virus (H5N1) infection in mice by inducing extensive inflammatory responses and severe pneumonia. The HMGB1 expression is induced in mice on days 3-7 post infection. The HMGB1-specific antibody can reduce the levels of inflammatory cytokines and chemokines and enhance the survival rate, but the virus titer is not affected by HMGB1 antibody treatment [51]. It was proposed that virus-mediated cytolysis after initial acute coronavirus infection of alveolar endothelial cells or macrophages results in HMGB1 release from the damaged cells. Extracellular HMGB1 would mediate deleterious pulmonary inflammatory responses, such as epithelial barrier derangement, neutrophil infiltration, lung edema and injury, which subsequently cause respiratory failure, and even death [52]. The global immune response analysis of pigs infected with PRRSV suggested that HMGB1 might manipulate the host immune response and eventually cause the immunosuppression in pigs [53]. Denguevirus infection induces a passive release of HMGB1 in A549 cells, which might also regulate TNF-α, IL-6, IL-8, and IFN-α secretion in DENV-infected DCs [34, 54]. Similar to the study on Dengue virus, our data also showed that HMGB1 was translocated from the nucleus into the cytoplasm and released into the extracellular space at the early stage of infection. Both PEDV infection and PEDV-N overexpression resulted in the increase of proinflammatory cytokines (TNF-α, IL-1β, IL-6, and IL-8), which could be attenuated by HMGB1 antibody treatment.

It has been shown that the capsid protein of Dengue virus is responsible for HMGB1 translocation from the nucleus to the cytoplasm, which is mediated by p300/CBP-associated factor (PCAF) acetylase complex [55]. In our studies, we also found that PEDV-N overexpression induced HMGB1 acetylation and release. HMGB1 acetylation and release induced by PEDV infection or PEDV-N overexpression could be attenuated by deacetylase Sirt1 activator and histone acetyltransferase inhibitor treatment, suggesting PEDV infection or PEDV-N could lead to the inhibition of deacetylase and activation of acetyltransferase while the exact mechanisms need further investigation.

Our studies further revealed that PEDV-N expression promoted HMGB1 transcription, which was associated with putative C/EBP binding motif in HMGB1 promotor region. In addition, co-immunoprecipitation experiment confirmed that PEDV-N interacted with the transcription factor C/EBP-β. The result is similar to the previous study on HTVL1, which shows viral protein Tax enhances the transcription of HMGB1 gene by interacting with C/EBP [41].

Although we found PEDV-N promoted HMGB1 acetylation/release and the increase of proinflammatory cytokines, we did not pursue the effect of viral RNA on HMGB1 and proinflammatory cytokine expression. It is well known that viral dsRNA promotes proinflammatory response through various pathways [56]. Our qRT-PCR result showed that virus infection resulted in significant increase of proinflammatory cytokines, which were higher than those in PEDV-N overexpression cells, suggesting that viral RNA or other viral proteins might also contribute to proinflammatory cytokines production.

Secreted HMGB1 can reduce DENV replication in DCs, accompanied with the increased expression of IFN-α [34]. Studies on HCV show that intracellular and extracellular HMGB1 play opposite roles in HCV propagation. Extracellular HMGB1 treatment may initiate antiviral responses to block HCV infection, whereas intracellular HMGB1 is important for HCV RNA replication [57, 58]. The opposite effects of HMGB1 have also been reported on HIV-1 virus [59, 60]. In the case of porcine reproductive and respiratory syndrome virus, recombinant HMGB1 does not affect virus replication in MARC-145 and PAM cells [35], suggesting virus infection blocked by HMGB1 might be virus-dependent. In our studies, we used Vero cells as virus infection model. Vero cells lack the IFN-α and IFN-β genes while interferon signal pathway is intact. Studies on DENV and HCV both suggested that extracellular HMGB1 reduces virus infection and triggers interferon response. Therefore, better cell culture model for PEDV is necessary to clarify whether released HMGB1 inhibits virus infection when IFN-α or IFN-β gene is present.

In summary, our studies demonstrated that PEDV infection led to the expression, acetylation and subsequent release of HMGB1. PEDV-N played active roles in these processes. PEDV-N increases the HMGB1 mRNA level by initiation of the gene transcription as a result of interaction with C/EBP-β. PEDV-N also promoted HMGB1 acetylation through histone acetyltransferase and NF-kB. Many studies reveal the essential roles of HMGB1 in modulating tissue damage and inflammation during sterile injury and infection [61]. In addition, extracellular HMGB1 also contributes to the pathogenesis of various chronic inflammatory and autoimmune diseases [21, 62–65]. All of these characteristics appoint HMGB1 as an important target in the treatment of multiple human diseases, including immune disorders, infectious diseases, and cancer. Our HMGB1 neutralization experiments confirmed its inhibitory effects on PEDV infection as well as on proinflammatory cytokine production, which provided a molecular basis for the development of therapeutic methods and drugs to control PED by modulating host immuno-inflammatory response

## MATERIALS AND METHODS

### Cell and virus

Vero cells (African green monkey kidney cell line) were cultured in high-glucose Dulbecco's modified Eagle's medium (DMEM, Invitrogen) supplemented with 10% newborn calf serum (16010-159, GIBCO) and 1% penicillin–streptomycin solution (Invitrogen). Porcine epidemic diarrhea virus (strain HLJBY, GenBank: KP403802.1) was propagated in Vero cells in DMEM supplemented with 2% newborn calf serum and 2.5 μg/mL of trypsin for 3 days [66]. The cells were frozen and thawed for 3 times, the media was centrifuged at 12,000×g for 10 min at 4°C. The supernatant was collected and stored at −80°C till use.

### Reagents and antibodies

Histone acetyltransferase inhibitor Garcinol (GAR), SIRT1 activator Resveratrol (RES), SIRT1 inhibitor Nicotinamide (NIC) and NF-κB inhibitor BAY 11-7082 (BAY) were obtained from Santa Cruz (Santa Cruz, CA). Antibodies against HMGB1 and β-actin were purchased from Cell Signaling Technology (Danvers, MA). Anti-acetyl lysine antibody (clone Kac-01) was purchased from PTM Biolab (Hangzhou, China). siRNAs were purchased from biotend (Shanghai, China). The bovine serum albumin (BSA) was purchased from Dingguo (Nanjing, China).

### Plasmid constructs

In order to construct HA-tagged viral N protein expressing plasmid, PEDV-N gene was first amplified using PCR with specific primers (Table 1) carrying *Not*I and *Xba*I restriction sites in forward and reverse primers. The PCR product was digested with *NotI* and *XbaI* and ligated to pCAGGS-HA (PCA) vector previously digested with these two enzymes. In order to construct HMGB1 luciferase reporter gene plasmids, HMGB1 promoter (GenBank: EF157968.1) fragments were first amplified by PCR with specific primers carrying *Kpn*I and *Bgl*II restriction sites in forward and reverse primers (Table 1). The PCR products were digested with *Kpn*I and *Bgl*II and ligated to pGL3-basic vector previously digested with these two enzymes. These reporter gene plasmids included pHLuc-HMGB1 (pHLuc1, −1622 to +83), pHLuc-HMGB1-1 (pHLuc2, −1264 to +83), pHLuc-HMGB1-2 (pHLuc3, −822 to +83), pHLuc-HMGB1-3 (pHLuc4, −382 to +83). The mutant luciferase reporter gene plasmids carrying mutations in transcription factor binding sites, including mLuc2-C (C/EBP), mLuc2-M (v-Myb), mLuc2-AP (AP-1), mLuc2-Cd (CdxA), and mLuc2-U (USF), were prepared with Mut Express® II Fast Mutagenesis kit (Vazyme, China) according to the manufacturer's instruction using plasmid pHLuc-HMGB1-1 as the template. The fidelity of the DNA constructs was verified by DNA sequencing.

**Table 1:** Sequences of the primers used for cloning and qRT-PCR

Name	Sequence
PEDV-N	Forward-GCACCGCGGCCGCATG GCT TCT GTC AGC TTT CAG G Reverse -GCACCTCTAGAATT TCC TGT GTC GAA GAT CTC G
pHLuc-HMGB1	Forward-GCACCGGTACCATAATCCCTGAGTCCGATAGTGTA Reverse-GCACCAGATCTTTATGCTCCTCCCGACAAG
pHLuc-HMGB1-1	Forward-GCACCGGTACCCCACTACAGCCAGCACATTT Reverse-GCACCAGATCTTTATGCTCCTCCCGACAAG
pHLuc-HMGB1-2	Forward-GCACCGGTACCGACATGCTGTGAAACTCGA Reverse-GCACCAGATCTTTATGCTCCTCCCGACAAG
pHLuc-HMGB1-3	Forward-GCACCGGTACCTTCGCTCCGGCGGCCGCTC Reverse-GCACCAGATCTTTATGCTCCTCCCGACAAG
PEDV-ORF3	Forward-TTTGCACTGTTTAAAGCGTCT Reverse-AGTAAAAGCAGACTAAACAAAGCCT
PEDV-N	Forward-GCACTTATTGGCAGGCTTTGT Reverse-CCATTGAGAAAAGAAAGTGTCGTAG
GAPDH	Forward-AGGTCGGAGTCAACGGATTT Reverse-TAGTTGAGGTCAATGAAGGG
IL-1β	Forward GGAAGACAAATTGCATGG Reverse CCCAACTGGTACATCAGCAC
IL-6	Forward AGAGGCACTGGCAGAAAAC Reverse TGCAGGAACTGGATCAGGAC
IL-8	Forward AGGACAAGAGCCAGGAAGAA Reverse ACTGCACCTTCACACAGAGC
TNF-α	Forward TCTGTCTGCTGCACTTTGGAGTGA Reverse TTGAGGGTTTGCTACAACATGGGC

### Western blotting analysis

The cells in 6-well plates (Corning) were washed with ice-cold PBS for three times. The cells were lysed with the lysis buffer (50mM Tris-HCl, pH 7.4, 150mM NaCl, 1% Triton X-100, 2mM EDTA, 0.1% SDS, 5mM sodium orthovanadate) containing the protease inhibitor cocktail tablet (Roche Molecular Biochemicals) and 0.1mM PMSF for 2 h at 4°C. The lysates were centrifuged at 12,000×g for 30 min at 4°C. Protein concentration was determined using the Bradford assay [67]. Equal amount of proteins (60μg) were subjected to sodium dodecyl sulfate poly-acrylamide gel electrophoresis (SDS-PAGE). The proteins in the gel were transferred to polyvinylidene fluoride (PVDF) membranes. The membranes were blocked with 3% BSA in PBS-T (PBS + 0.05% Tween-20, pH 7.4) at 4°C for 16h, followed by incubation for 2 h with appropriate primary antibody (anti-HMGB1, anti-acetyl lysine, anti-HA, anti-β-actin or anti-PEDV-N). The membranes were washed three times with PBS-T before incubated with the appropriate secondary antibody (HRP-anti-rabbit IgG or HRP-anti-mouse IgG) for 1 h. The immunoreactive bands were visualized by an ECL enhanced chemiluminescence system (Biouniquer, China).

### qRT-PCR

Total RNA was extracted and purified from the Vero cells with TRIzol reagent (Invitrogen) according to the manufacturer's recommendations. The cDNA were synthesized from 1 μg of total RNA using a HISCRIPT 1st strand cDNA synthesis kit (Vazyme, China). The quantitative real-time PCR was performed using SYBR Green Master Mix according to the manufacturer's protocol (Vazyme, China). The primer sequences for IL-1β, IL-6, IL-8, TNF-α, ORF3 of PEDV and GADPH are listed in Table 1. GAPDH was as the internal control. The relative expression levels of the genes were compared with those of GAPDH by the 2^−ΔΔCt^ method. The qRT-PCR experiments were performed with CFX96 Real-time PCR system (Bio-Rad). Each assay repeated in triplicate.

### Immunofluorescence staining and confocal microscopy

Vero cells were first seeded on coverslips. The cells were then infected with PEDV (MOI = 0.1) for 6h at 37°C. The cells were fixed with 4% formaldehyde for 15 min at room temperature, permeabilized with 0.1% Triton X-100 for 10 min and incubated with 5% BSA for 1h. The cells were incubated with HMGB1 antibody for 2h at 37°C before incubated with Alexa Fluor 488-conjugated anti-rabbit IgG for 0.5h at 37°C. After PBS-T wash, the cells were incubated with PEDV-N antibody for 2h at 37°C before incubated with Alexa Fluor 555-conjugated anti-mouse IgG for 0.5h at 37°C. The nuclei were stained with 4′-6-diamidino-2-phenylindole (DAPI, Dinguo, China). Fluorescent images were obtained under a fluorescence microscope (Leica).

To study the colocalization of PEDV-N and C/EBP-β or NF-κB, Vero cells were transfected with pCAGGS-HA-N (PCA-N) for 24h. The cells were fixed with 4% formaldehyde for 15 min at room temperature, permeabilized with 0.1% Triton X-100 for 10 min and incubated with 5% BSA for 1h. The cells were incubated with either C/EBP-β or NF-κB antibody for 2h at 37°C before incubated with Alexa Fluor 488-conjugated anti-rabbit IgG for 0.5h at 37°C. After PBS-T wash, the cells were further incubated with PEDV-N antibody for 2h at 37°C followed by incubation with Alexa Fluor 555-conjugated anti-mouse IgG for 0.5h at 37°C. The nuclei were stained with 4′-6-diamidino-2-phenylindole (DAPI, Dinguo, China). Confocal images were obtained using a Zeiss LSM 710 scanning confocal microscope.

### Plaque formation assay

Virus culture supernatant with 10 fold dilutions (from 10^2^ to 10^5^) were added into 6-well plates with the confluent monolayer of Vero cells. The excess virus inocula were removed by PBS wash 1h later. Subsequently, overlay medium (2.5% low melting point agarose with DMEM medium containing 4% newborn calf serum) was added to each well and the plates were further incubated at 37°C with 5% CO_2_ for 3 days. The cells were stained with 0.5% crystal violet.

### RNA interference

Vero cells were grown to 50–60% confluence in 6-well cell culture plates and then transiently transfected with indicated small interfering RNA (siRNA) using polyethylenimine (PEI) [68]. The silence efficiency of the siRNA was detected by western blotting analysis and qRT-PCR.

### Chromatin immunoprecipitation (CHIP)

The experiments were performed according to previous reports [69, 70]. 1×10^6^ cells were cross-linked with 1% formaldehyde for 10 min at room temperature. The reaction was quenched and washed with PBS for three times. Cells were scraped with PBS, and centrifuged at 2000×g for 4 min at 4°C. The cells were lysed with warm SDS lysis buffer for 10 min on ice. Cells were sonicated for 1min (sonication for 3s pause for 7s, total 20 times) to shear DNA to lengths between 200 and 1000 bp on ice. Subsequently, the sonicated chromatin was incubated for 16h with the anti-N polyclonal antibody or anti-HA monoclonal antibody and protein G beads. The beads were washed with washing buffer (50mM Tris-Cl, pH7.4, 150mM NaCl, 0.1% SDS, 1% sodium deoxycholate and 1% Triton-X100). PCR was performed with the primers specific for HMGB1 gene: 5'-CCACTACAGCCAGCACATTT-3' (sense), 5'-TAGATGCAGGCAGAGAGTGC-3' (anti-sense).

### Reporter gene assay

To explore how PEDV-N regulated HMGB1 transcription in Vero cells, the cells were plated in 24-well plates at a density of 1×10^5^ cells/well. The cells were co-transfected with 100ng luciferase reporter plasmids (pHLuc-HMGB1, pHLuc-HMGB1-1, pHLuc-HMGB1-2 or pHLuc-HMGB1-3), 200ng PCA-N or control plasmid PCA, and 10ng renilla luciferase reporter plasmid pRL-TK. Luciferase activity was measured 24h after the transfection with a dual-luciferase assay system (TransGen Biotech).

To determine the binding site of the transcription factor, Vero cells were co-transfected with 100ng mutant reporter gene plasmid (mLuc2-C, mLuc2-M, mLuc2-AP, mLuc2-Cd or mLuc2-U) or WT plasmid (pHLuc2), 200ng PCA-N or PCA and 10ng renilla luciferase reporter plasmid pRL-TK for 24h. Luciferase activity was determined with a dual-luciferase assay system (TransGen Biotech). Data represented relative firefly luciferase activity normalized to renilla luciferase activity.

### Co-immunoprecipitation (Co-IP)

Co-IP was performed as previously described [71]. Briefly, PEDV infected Vero cells were lysed with the lysis buffer (50mM Tris-HCl, pH8.0, 150mM NaCl, 1mM EDTA, 1% NP-40) containing DNase (100μg/ml) and RNase A (100μg/ml). The supernatant of cell lysate was pre-cleared with protein G for 4h at 4°C (Invitrogen). After centrifugation, the supernatant was then divided into two parts; one part was incubated with anti-N antibody or anti-HA antibody mixed with protein G while the other part was incubated with anti-rabbit IgG mixed with protein G at 4°C overnight. After extensive washes, immunoprecipitates were analyzed by western blot.

### Cytotoxicity assay

Approximately 2×10^4^ Vero cells per well were seeded in a 96-well cell culture plate and cultured for 24 h at 37°C in the presence of 5% CO_2_. The medium was replaced with fresh DMEM supplemented with 2% newborn calf serum containing inhibitors and the plates were incubated for up to 24 h. The cytotoxicity was assayed by measuring lactate dehydrogenase (LDH) released from the cells using Cytotox-One homogenous membrane integrity kit (Promega, USA) according to the manufacturer's instructions. The cytotoxicity experiment showed that resveratrol, nicotinamide, garcinol and BAY 11-7082 did not cause significant cytotoxicity at the working concentrations (data not shown).

### Statistical analysis

All data were determined in triplicate and are representative of at least two separate experiments. The results represent the means ± standard deviations of triplicate determinations. The differences between means were considered significantly at * p < 0.05, very significant at ** p < 0.01. All statistical analyses were performed by one-way ANOVA using a SPSS 17.0 software package (version 16.0, SPSS Inc., Chicago, IL, USA).
